# Molecular typing of *Mycobacterium tuberculosis* isolates circulating in Henan, central China

**DOI:** 10.3892/etm.2012.699

**Published:** 2012-09-06

**Authors:** YULING ZHAO, HUI LI, JIN XING, HONGYI YANG, XIAOGUANG MA, JIYING XU, JIE SHI, GUORUI YAN

**Affiliations:** Center for Disease Control and Prevention of Henan Province, Zhengzhou, Henan 450016, P.R. China

**Keywords:** genotyping, spoligotyping, Beijing family

## Abstract

The aim of this study was to characterize the genotypes of *Mycobacterium tuberculosis* (MT) in isolates obtained from Henan, China, and to study the distribution features of Beijing strains in Henan. A total of 443 MT strains isolated in Henan Province were tested for susceptibility to isonicotinylhydrazide (INH), rifampicin (RFP), ethambutol (EMB) and streptomycin (SM), and genotyped by spoligotyping. The clustering of genotypes revealed 4 gene clusters (Beijing and Beijing-like, T, Manu, and S and LAM3) and 24 genotypes. In total, 387 (87.4%) of the strains were Beijing strains. The frequency of multidrug-resistant (MDR) strains was significantly higher in the Beijing and Beijing-like strains than in the other strains (χ^2^=4.6564, P<0.05). However, the percentages of drug resistance and sensitivity in the Beijing strains were almost the same as those in the non-Beijing strains. The proportion of Beijing strains in the ≤60-year-old TB patients was significantly higher than in the >60-year-old TB patients (χ^2^=32.053, P<0.001). The distribution of Beijing strains deceased gradually from the east to the west in Henan Province (P<0.0001). The data demonstrate that the Beijing genotype is a major type in the area and may be related to enhanced transmissibility. The transmission of Beijing family strains has increased in Henan and its incidence is highest in the east of Henan. The MDR strains were significant in the spread of MT.

## Introduction

*Mycobacterium tuberculosis* (MT) is seriously harmful to human survival and health. Currently it constitutes the second major cause of mortality due to infectious disease worldwide. The scientific challenge in MT control has become increasingly complicated with the emergence of new more severe forms of tuberculosis: extensively drug-resistant tuberculosis (XDR-TB) and human immunodeficiency virus (HIV)-TB co-infection (1). The rapid and accurate identification of MT is important for controlling the spread of tuberculosis (TB).

With the rapid development of molecular biology and technology, numerous genotyping methods have been established for specific nucleic acid sequences. Spoligotyping is a rapid, polymerase chain reaction (PCR)-based method for genotyping strains of the MT complex. Its design was reported by Kamerbeek *et al* in 1997 (2). Spoligotyping data may be represented digitally; therefore, the results may be readily shared among laboratories. With these advantages, spoligotyping has been widely applied to identify MT strains worldwide (3,4). Spoligotyping uses a highly variable set of signaling characteristics to obtain phylogenetic information. Thereby, Sola *et al* established a TB polymorphism database in 2001 (5). The 4th international spoligotyping database, SpolDB4, describes 1939 shared types (STs) of MT, representing a total of 39,295 strains from 122 countries. These strains have been tentatively classified into 62 clades and/or lineages using a mixed expert-based and bioinformatics approach (6). The prevalence of MT may relate to the specific genotype family, identified by genotyping methods. Different genotypes have unique molecular characteristics, geographical distributions and pathogenicities (7,8). From a genotype study of 3,000 strains of MT obtained from 19 countries, in 1995 van Soolingen *et al* found that the MT Beijing genotype family has genetic characteristics that are unique and significant (7). The MT Beijing strain is widely distributed in Far East Asia and approximately 86% of clinical isolates in China belong to this family (3). This finding has had a major impact on the epidemiological study of MT. The spread of the MT Beijing strain has been reported in other countries in Asia and Eastern Europe, particularly Russia (5,8–15). Currently, MT Beijing strains are identified principally by the number of spacers in the direct-repeat (DR) region of the MT genome, which is characterized by the deletion of spacers 1–34 and the presence of most of the spacers 35–43. In addition, certain MT Beijing-like genotypes are characterized not only in terms of deletions of spacers 1–34 but also by certain individual deletions in spacers 35–43.

Henan Province is located in central China and in the middle and lower reaches of the Yellow River. The primary part of Henan is located to the south of the Yellow River. The total area of Henan is 167,000 km^2^, accounting for 1.74% of the total area of China. Henan has a total population of 98.69 million living in 109 counties, among which 31 are national poverty counties and 13 are provincial poverty counties. The population of migrant workers in Henan is huge with great mobility.

The MT Beijing family strains account for 13% of global isolates of MT and cause approximately 33% of tuberculosis infections worldwide (16,17). However, the reason that the MT Beijing family has spread so prolifically compared with other strains remains unknown. Studies from animal models have shown that it may be due to its ability to bypass Bacillus Calmette-Guérin (BCG) immunization (18). A large number of epidemiological studies have investigated the correlation between the MT Beijing family and drug resistance. These studies have reached 4 conclusions: MT Beijing is endemic and is not related to drug resistance; MT Beijing is widely prevalent and drug resistant; MT Beijing is widespread and drug sensitive; and MT Beijing has low levels of drug resistance. The differences in findings may be attributable to the various technologies and different drugs used in these studies (18) and/or the different subfamilies of the MT Beijing strains, which have different properties (2). Therefore, it is important to continue to investigate the correlation between MT Beijing strains and drug resistance, which may help to reveal the reasons behind its prevalence in Henan Province. Henan spans the middle and lower reaches of the Yellow River. The high prevalence of multidrug-resistant (MDR)-TB among the TB patients in Henan Province has been a major challenge for MT control. According to a report of the national drug resistance baseline (2007–2008) edited by Ministry of Health of China, the rates of primary and acquired MDR-TB were 5.71 and 25.64%, respectively. Using spoligotyping technology, we have determined that the MT Beijing family is the main genotype of MT circulating in Henan Province. To determine the reasons for its prevalence, we conducted drug-sensitivity experiments.

## Materials and methods

This study was approved by the Institutional Review Board of the Center for Disease Control and Prevention of Henan Province (China) and written informed consent was obtained from each participant.

### MT strains

A total of 443 isolates of *MT* were included in this study. The isolates were obtained from projects that were surveyed for the national drug resistance baseline for TB and from clinical patients from the Henan province in 2009. Samples were cultured and isolated with regular Löwenstein-Jensen (L-J) medium to obtain positive cultures, which were then kept at −80°C. The standard MT strain, H37Rv, was used as the control; it was provided by the National Tuberculosis Reference Laboratory of the Chinese Center for Disease Control and Prevention, Beijing, China.

### Identification of drug resistance

Strain identification and drug-susceptibility testing were performed according to World Health Organization/International Union Against Tuberculosis and Lung Disease (WHO/IUATLD) guidelines (19). A total of 344 strains were tested for susceptibility to 4 drugs: isonicotinylhydrazide (INH, 0.2 μg/ml), rifampicin (RFP, 40.0 μg/ml), ethambutol (EMB, 2.0 μg/ml) and streptomycin (SM, 4.0 μg/ml). Positive cultures were defined by a ratio of resistant clones in drug-containing medium to control medium of >1%.

### Preparation of DNA of MT

Collected isolates were inoculated in L-J medium at 37°C for 2–4 weeks. A loop of colonies was placed in 500 μl Tris-ethylenediamine tetraacetic acid (EDTA) buffered solution (TE), inactivated at 80°C for 30 min. The mixture was then treated in boiling water for 10 min. The supernatants were cleared of large debris by centrifugation at 12,000 rpm for 2 min and were subsequently kept at −20°C.

### Spoligotyping technology

Hybrid membranes of spoligo-typing, mini-blotter sample-board and biotin-labeled primers DRa and DRb (DRa: 5′-GGT TTT GGG TCT GAC GAC-3′, DRb: 5′-CCG AGA GGG GAC GGA AAC-3′) were purchased from Isogen Life Science (De Meern, Netherlands). The streptavidin-POD (peroxidase)-conjugate and chemiluminescence (ECL) detection system were purchased from Roche Applied Science (Mannheim, Germany). All steps were performed according to the instructions provided by Isogen. Detailed procedures were described previously by Kamerbeek *et al* (2). The DNA of MT was firstly amplified by primers DRa and DRb and then hybridized with membranes comprising 43 oligonucleotide probes (Table I).

### PCR reaction system

Upstream and downstream primers, dNTP, buffer for Taq, Taq and DNA template were mixed together and added to 50 μl double-distilled water. PCR was performed using Taq polymerase under standard conditions at 96°C for 3 min, then at 96°C for 1 min, 55°C for 1 min and 72°C for 30 sec. This procedure was repeated for 30 cycles and finally the mixture was kept at 72°C for 10 min.

### Membrane hybridization

The labeled membrane was transferred to a mini-blotter sample-board. The PCR product was then added and the board was incubated at 60°C for 60 min. The membrane was then washed at 60°C with 2X SSPE/0.5% SDS for 10 min, followed by incubation with l2X SSPE/0.5% SDS containing 2.5 μl streptavidin-biotin at 42°C for 60 min. Finally, the membrane was washed twice with 2X SSPE/0.5%SDS at 42°C for 60 min and twice with 2X SSPE for 5 min.

### Chemiluminescence detection of hybrid DNA

The membrane was incubated with an ECL detection system for 1 min and then covered with a transparent plastic film. The membrane was then placed into a dark cassette and exposed to X-ray film.

### Epidemiological data

Epidemiological data included the age of the patients and the sources of the strains. A total of 443 isolates were collected from 2007 to 2009; 334 were from >60-year-old patients and 109 were from ≤60-year-old patients.

### Statistical analysis

Genotype data were analyzed using the MIRU-VNTRplus web-based application (http://www.miru-vntrplus.org/MIRU/index.faces). Statistical data were analyzed using the χ^2^ test. P<0.05 was considered to indicate a statistically significant result.

## Results

### Beijing family strains are the most common genotype of MT in Henan

**Figure 1:**
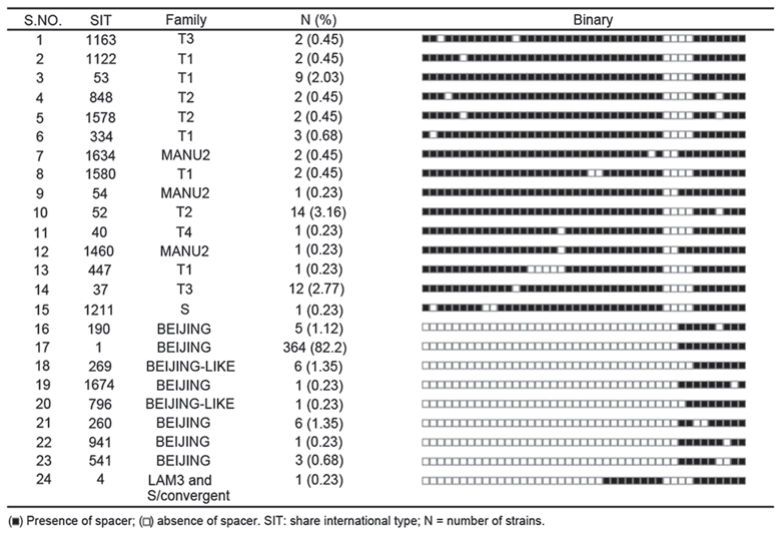
Genotypes of *Mycobacterium tuberculosis* in Henan.

The MT strains were genotyped by spoligotyping, and then the genotype clusters were analyzed using the MIRU-VNTRplus web-based application. The results revealed that the 443 strains may be categorized into 4 MT gene clusters (Beijing and Beijing-like, T, Manu, and S and LAM3) and 24 genotypes (Fig. 1). The 387 MT Beijing strains accounted for 87.4% of the TB cases recorded in Henan, T type accounted for 11.3%, Manu type accounted for 0.9%, S and LAM3 accounted for 0.45% (Table II).

### Multidrug resistance is significantly higher in the MT Beijing strains than in the non-Beijing strains

In addition to determining genotype clusters, drug sensitivity tests were performed on each of the 443 strains studied. Of these, 387 strains were Beijing-type MT, and of these, 30 were MDR, accounting for 7.8% (Table III). However, no MDR strain was found among the 56 non-Beijing strains studied. Significant differences were found between the 2 families using the χ^2^ test (χ^2^=4.5570, P<0.05; Table III). Our results revealed that 12 (27%) of the MDR strains were resistant to INH + RIF + SM + EMB, 10 (2.3%) were resistant to INH + RIF + SM, 6 (1.4%) were resistant to INH + RIF and 2 (0.5%) were resistant to INH + RIF + EMB. Overall, 73.9% of the MT Beijing strains and MT Beijing-like strains were sensitive to drugs. The percentage is similar to that in the non-Beijing strains. Also, we observed no differences in any drug resistance between the MT Beijing and non-Beijing strains.

### Age significantly correlates with the distribution of the Beijing strains

To further investigate the cause of the widespread MT Beijing isolates in Henan, we assessed the distribution of Beijing strains in different age groups (Table IV). Notably, the age of 60 years formed a very clear boundary. Beijing strains accounted for 94.5% of isolates from ≤60-year-old patients but only 67.7% of those from >60-year-old patients.

### Geography significantly correlates with the distribution of Beijing strains

To determine the distribution MT Beijing isolates in Henan Province, we classified TB according to geographic location. The proportion of Beijing isolates was then determined (Table V). Beijing strains accounted for 97.1% of TB cases in Fugou County in the eastern part of Henan Province. The rate of MT Beijing in Xinmi was similar to that in Zhongmo; Xinmi and Zhongmo are located in the central part of Henan Province. MT Beijing isolates accounted for 88.9% of TB cases in Whishi, also in eastern Henan. In Nanyang, which is located in the most southern part of Henan, Beijing strains accounted for 87.6% of TB cases. MT Beijing isolates accounted for 67.6% of cases in Song County in the most western part of Henan. These data reveal that the distribution of the MT Beijing genotype family extends from the eastern to the central areas of Henan and also from the southern end to the western edge. This is the first report of these characteristics of MT Beijing in Henan.

In this study, we typed 443 isolates of MT in Henan Province using spoligotyping technology. These data elucidated the epidemiology of MT in the region. In total, 443 isolates were collected from TB patients in six counties. Multiple strains were recorded in each county: Song County (68 strains), Zhongmo (63 strains), Xinmi (71 strains), Fugou (69 strains), Nanyang (73 strains) and Weishi (99 strains).

## Discussion

The MT Beijing genotype family represented 87.4% of the total TB cases in Henan Province. This rate is higher than in Ho Chi Minh City, Vietnam (53%) and Hanoi (58%) (20). MDR-TB accounted for 7.8% of the MT Beijing strains, but multidrug resistance did not emerge in the non-Beijing families, in types T1, T2, T3, T4 and other types. Therefore, the monitoring of the MT Beijing isolates should be focused on the surveillance of MDR strains.

Age significantly correlated with the distribution of the Beijing family strains (Table IV). The proportion of Beijing isolates in the ≤60-year-old group was significantly higher than in the >60-year-old group. Additional studies of the transmission characteristics in different age groups are urgently required. Although we were not able to determine the detailed proportions of recent transmission exactly, these data suggest that the present TB patients were subject to recent transmission. Dou *et al* found that MT Beijing isolates accounted for 52.5% of cases in a genotyping study of 356 cases of MT in Taipei (21). In their study, Beijing family isolates that emerged more recently were found to have greater multidrug resistance and to be more prevalent in younger individuals. The more recent strains of MT Beijing accounted for 85.3% of TB cases in patients aged <25 years, which suggests that this strain is likely to become prevalent in Taipei. Since the results of this study are similar to those of the current study, it is important that we continue with our investigations.

Finally, we found that geography also significantly correlated with the distribution of the MT Beijing genotypes. Distribution of the MT Beijing family spanned the eastern to central parts of Henan Province as well as the southern end to the westernmost area. The varying distribution of MT Beijing among these regions is worthy of note and its causes require further investigation. In a study of TB samples isolated from 22 regions in China, Liu *et al* found that MT Beijing strains in northern China (83.91% of all TB cases) accounted for a significantly higher proportion of cases than in the southern region (66.4% of all TB cases) (22). This result suggests that subfamilies of Beijing strains may exist in different regions. Our preliminary study indicated that Beijing strains may spread from east to west across the Henan Province. Further studies are required to verify this theory.

To our knowledge, this is the first genotyping study of MT using spoligotyping in Henan. Our data demonstrate that the most common genotype of MT in Henan is the MT Beijing family. Multidrug resistance correlated with the prevalence of the Beijing family in the area. The proportion of MT Beijing strains was higher in younger age groups, suggesting that it has spread more rapidly recently. Finally, our preliminary study showed that MT Beijing strains may spread from east to west across the Henan Province.

## Figures and Tables

**Table I:** Oligonucleotide probes used in this study.

Space no.	Oligonucleotide sequence (primer 5′-3′)
1	ATAGAGGGTCGCCGGTTCTGGATCA
2	CCTCATAATTGGGCGACAGCTTTTG
3	CCGTGCTTCCAGTGATCGCCTTCTA
4	ACGTCATACGCCGACCAATCATCAG
5	TTTTCTGACCACTTGTGCGGGATTA
6	CGTCGTCATTTCCGGCTTCAATTTC
7	GAGGAGAGCGAGTACTCGGGGCTGC
8	CGTGAAACCGCCCCCAGCCTCGCCG
9	ACTCGGAATCCCATGTGCTGACAGC
10	TCGACACCCGCTCTAGTTGACTTCC
11	GTGAGCAACGGCGGCGGCAACCTGG
12	ATATCTGCTGCCCGCCCGGGGAGAT
13	GACCATCATTGCCATTCCCTCTCCC
14	GGTGTGATGCGGATGGTCGGCTCGG
15	CTTGAATAACGCGCAGTGAATTTCG
16	CGAGTTCCCGTCAGCGTCGTAAATC
17	GCGCCGGCCCGCGCGGATGACTCCG
18	CATGGACCCGGGCGAGCTGCAGATG
19	TAACTGGCTTGGCGCTGATCCTGGT
20	TTGACCTCGCCAGGAGAGAAGATCA
21	TCGATGTCGATGTCCCAATCGTCGA
22	ACCGCAGACGGCACGATTGAGACAA
23	AGCATCGCTGATGCGGTCCAGCTCG
24	CCGCCTGCTGGGTGAGACGTGCTCG
25	GATCAGCGACCACCGCACCCTGTCA
26	CTTCAGCACCACCATCATCCGGCGC
27	GGATTCGTGATCTCTTCCCGCGGAT
28	TGCCCCGGCGTTTAGCGATCACAAC
29	AAATACAGGCTCCACGACACGACCA
30	GGTTGCCCCGCGCCCTTTTCCAGCC
31	TCAGACAGGTTCGCGTCGATCAAGT
32	GACCAAATAGGTATCGGCGTGTTCA
33	GACATGACGGCGGTGCCGCACTTGA
34	AAGTCACCTCGCCCACACCGTCGAA
35	TCCGTACGCTCGAAACGCTTCCAAC
36	CGAAATCCAGCACCACATCCGCAGC
37	CGCGAACTCGTCCACAGTCCCCCTT
38	CGTGGATGGCGGATGCGTTGTGCGC
39	GACGATGGCCAGTAAATCGGCGTGG
40	CGCCATCTGTGCCTCATACAGGTCC
41	GGAGCTTTCCGGCTTCTATCAGGTA
42	ATGGTGGGACATGGACGAGCGCGAC
43	CGCAGAATCGCACCGGGTGCGGGAG

**Table II:** Genotyping results.

Strains	Proportion	Strain no.
Beijing and Beijing-like	87.4%	387
T (T1, T2, T3, T4)	11.3%	50
Manu	0.9%	4
S and LAM3	0.45%	2

**Table III:** Differences in the characteristics between MT Beijing (n=387) and non-Beijing (n=56) family strains.

Factors	Beijing n (%)	Non-Beijing n (%)	χ^2^	P-value
Gender				
Male	275 (71.0)	35 (62.5)		
Female	112 (28.9)	21 (37.5)	1.7060	0.1915
Pan-susceptible	286 (73.9)	44 (78.6)		
Drug-resistance	71 (18.3)	12 (21.4)	0.0715	0.7891
MDR-TB				
INH + RIF	6 (1.4)			
INH + RIF + SM	10 (2.3)			
INH + RIF + EMB	2 (0.5)			
INH + RIF + SM + EMB	12 (2.7)			
Total MDR	30 (7.8)		4.5570	0.0328

MT, *Mycobacterium tuberculosis*; MDR, multidrug-resistant; INH, isonicotinylhydrazide; RIF, rifampicin; SM, streptomycin; EMB, ethambutol.

**Table IV:** Distribution of the Beijing genotype by age group.

Age (Years)	Beijing strains n (%)	Odds ratio	95% CI	P-value
<25	85 (96.6)	1	-	
26–40	110 (94.5)	0.97	0.21–4.45	1.00
41–60	125 (94.6)	0.90	0.21–3.85	1.00
61–75	44 (67.7)	0.06	0.02–0.23	<0.0001
>76	23 (52.3)	0.04	0.01–0.14	<0.0001

CI, confidence interval.

**Table V:** Distribution of the Beijing genotype by region.

Region	Beijing strains n (%)	Pearson’s χ^2^ value	P-value
Song County	46 (67.6)		
Zhongmo County	57 (90.5)		
Xinmi County	65 (91.5)	31.76	<0.0001
Fugou County	67 (97.1)		
Nanyang County	64 (87.6)		
Weishi County	88 (88.9)		
